# Qubit-Programmable Operations on Quantum Light Fields

**DOI:** 10.1038/srep15125

**Published:** 2015-10-15

**Authors:** Marco Barbieri, Nicolò Spagnolo, Franck Ferreyrol, Rémi Blandino, Brian J. Smith, Rosa Tualle-Brouri

**Affiliations:** 1 Dipartimento di Scienze, Università degli Studi Roma Tre, Rome, Italy; 2 Dipartimento di Fisica, Sapienza Università di Roma, Rome, Italy; 3 Laboratoire Photonique, Numérique et Nanostructures, Institut d’Optique, CNRS and Université de Bordeaux, Talence, France; 4 Centre for Quantum Computation and Communication Technology, School of Mathematics and Physics, University of Queensland, St Lucia, Queensland 4072, Australia; 5 Clarendon Laboratory, Department of Physics, University of Oxford, United Kingdom; 6 Laboratoire Charles Fabry, Institut d’Optique, CNRS and Université Paris-Sud, Palaiseau, France and Institut Universitaire de France, Paris, France

## Abstract

Engineering quantum operations is a crucial capability needed for developing quantum technologies and designing new fundamental physics tests. Here we propose a scheme for realising a controlled operation acting on a travelling continuous-variable quantum field, whose functioning is determined by a discrete input qubit. This opens a new avenue for exploiting advantages of both information encoding approaches. Furthermore, this approach allows for the program itself to be in a superposition of operations, and as a result it can be used within a quantum processor, where coherences must be maintained. Our study can find interest not only in general quantum state engineering and information protocols, but also details an interface between different physical platforms. Potential applications can be found in linking optical qubits to optical systems for which coupling is best described in terms of their continuous variables, such as optomechanical devices.

Control of quantum systems is a key task for any implementation of quantum technologies, as well as for fundamental quantum physics tests^1^. Quantum optics, despite the fact that nonlinear interactions are extremely challenging to achieve for few-photon-level quantum light, has made considerable progress thanks to the adoption of measurement-induced nonlinearities^2^. This technique for inducing nonlinear behaviour in otherwise linear systems consists in utilising ancillary resources, and then perform conditional operations based on the outcome of a measurement on a part of the whole system. It has been primarily applied to the implementation of quantum gates for qubits encoded in single-photon degrees of freedom^3^,^4^,^5^,^6^,^7^,^8^.

Recent developments have shown that the same idea can be exploited for more elaborate control, when it is the state of the quantum field itself that is manipulated through heralding measurements^9^,^10^. This is the case, for instance, of the operation of quantum gates in coherent-state quantum computing^11^,^12^,^13^,^14^, entanglement distillation^15^,^16^, photon addition and subtraction^17^,^18^,^19^ related to the production of finite-dimensional quantum states^20^,^21^, and noiseless amplification^22^,^23^,^24^,^25^. A new hybrid approach has built up from these investigations that aims at merging the advantages of a continuous-variable approach to quantum optics, with experimental and conceptual tools proper to single-photon manipulation, and viceversa^26^,^27^,^28^,^29^,^30^.

In this paper, we propose a different kind of interface between these two approaches in the concept of qubit-programmed operations on a quantum field. Our proposal extends current methods for implementing photon addition and subtraction to operate with an arbitrary superposition, whose parameters can be set conditionally on the logical state of a qubit, encoded in a degree of freedom of a single photon. In principle, our scheme can be embedded in a larger architecture: the program can be determined as the result of a former quantum computation, thus allowing the possibility of it being in a superposition state. The information processing is initially carried out on discrete variables, but it affects the state of the continuous variables quantum field. This solution is particularly appealing in that the program state can be prepared with high fidelity due to the current available methods for the manipulation of discrete-variable systems, including entangled states. These properties add new capabilities to the state engineering toolbox for quantum technologies.

## Results

### Qubit-controlled operations on continuous-variable fields

**Figure 1:**
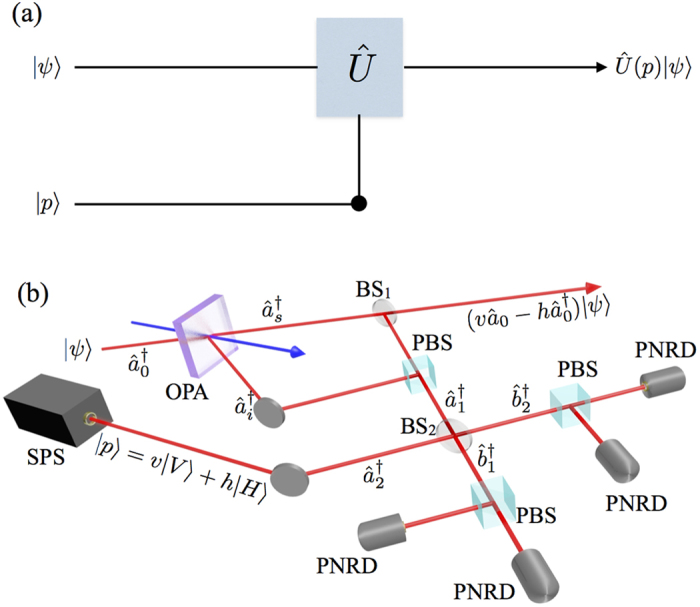
(**a**) Conceptual diagram of the programmable gate; (**b**) Experimental schematic of the programmable device indicating different interfering modes. BS_1_ has transmission coefficient *t* ~ 1. Output modes of the OPA and the single-photon source (SPS) are conveyed on the symmetric beamsplitter BS_2_. Polarisation-resolved detection is performed by means of photon-number resolving detectors (PNRDs).

The general idea is illustrated in Fig. 1a: a quantum field, described by a state 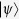, is the target we aim at manipulating through an operation 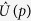, set according to the instructions *p* we received from a second party; in other words, these instructions represent a programme that configures a particular choice of 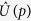. In the most general case, 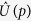 will be represented as a coherent superposition of some elementary operations. Therefore, the programme will need to be in the form of a quantum state 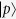, so that a proper mapping can be applied. Differently from conditional gates, such as the controlled-NOT gate for two-qubits, we do not require that the programme state is preserved. Analogic control has been implemented using an optical displacement to modulate the probability amplitude of single-photon subtraction^11^,^13^,^31^, and extensions to optomechanics have been discussed^32^; here we analyse a scheme for implementing the interface between a quantum optical field and the simplest discrete programme, a qubit^33^.

At the basis of our proposal, there is the possibility of realising photon subtraction by means of a simple high-transmissivity beamsplitter, and photon addition by using an optical parametric amplifier (OPA) in the low-gain regime. These operations can only be implemented probabilistically, with a single photon acting as a herald: in the former case, this comes from the reflected mode, in the latter, from the idler mode of the OPA. It is also known that these events can be made indistinguishable by quantum interference, thus erasing the information as to whether the trigger signal originated from an addition or subtraction heralding photon^34^,^35^,^36^. Inspired by the setup for quantum teleportation, we can use a single photon to control the degree to which the erasure of information occurs. We notice that previous schemes for quantum state engineering, notably quantum scissors^20^,^21^, have made use of similar concepts, using a split single photon as the entangled resource.

### Details of the protocol

Figure 1b details the apparatus of our proposal: consider the spatial mode 0 prepared in the *n*-photon Fock state 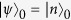, which constitutes the input to an OPA, driven in such conditions that well approximate photon addition. In terms of the two-mode interaction 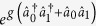, this implies working in the regime of low parametric gain *g* ≪ 1. Further to the action of the OPA, the state is then passed through a high-transmissivity mirror (*t*^2^ ~ 1, *r*^2^ ≪ 1) that realises photon subtraction. The two heralding modes are superposed on the spatial mode 1, using two orthogonal polarisations: horizontal (H) for the subtraction herald, vertical (V) for the addition. The action of this device on 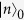 gives the following expression for the output state:
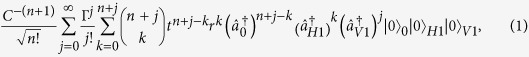
obtained by invoking the canonical beam splitter transformations, as well as the disentangling theorem^37^. Here, we have used the notation *C* = cosh *g*, Γ = tanh *g*, with *g* the parametric gain, i.e. squeezing parameter.

Due to the correlations established between modes 0 and 1, any detection on the latter results in an effective operation applied to the input field; as an example, the detection of a single photon on mode 1 on the polarisation *H* (*V*) would herald realisation of photon subtraction (addition). Detection in the diagonal polarisation basis would erase the information about the origin of the photon. Thus the state on mode 0 is transformed with the equal superposition 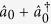, and similarly for other choices of polarisation states, which can be readily obtained by suitable choice of optical elements^34^,^35^.

**Figure 2:**
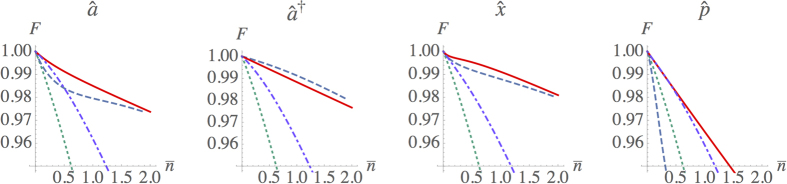
Fidelity *F*(*ρ*_*out*_, *ρ*_*target*_(*n*)) of the output states *ρ*_*out*_, as a function of the average photon number 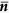 for four choices of the operator 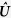, and different families of input states *ρ*_*in*_(*n*): coherent states 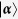 with *α* real solid red), cat states 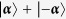 (dashed blue), single-mode squeezed vacuum (dotted green). We also include the case when *U* acts on a half of a two-mode squeezed state (dot-dashed purple). The transmission coefficient of the subtraction beamsplitter is *t* = 0.95.

Our aim is to control the choice of superposition by programming it on a qubit in the form 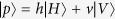, *i.e* in the ‘dual-rail’ encoding: 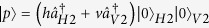. To achieve our purpose, we herald the two optical modes 1 and 2 on a beamsplitter with transmission coefficient 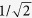, and then herald on either the outcome 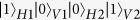 or 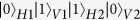. In an ordinary teleportation experiment, this would correspond to selecting the singlet in a Bell-state analysis. Consequently, the state of mode 0 is projected to:
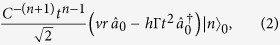
therefore, in the limit *t* ~ 1 and tuning the gain of the OPA so that Γ = *rt*^−2^, we can map the state of the qubit 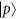 onto the heralded operator 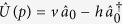 that acts on the input; detailed calculations are reported in the Supplementary Information. Based on the transformation Eq. (2), we can calculate the fidelity of the output states with the ideal case for different families of inputs. The results for four relevant cases 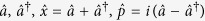, with a transmission *t* = 0.95, typical in such experiments^11^,^14^,^38^,^39^, is reported in Fig. 2.

### Analysis of the process fidelity

While a reasonable level of fidelity can be reached at low photon numbers for different relevant classes of input states, the precise behaviour with respect to the ideal process operation depends on the specific class of input state. We can highlight two general observations: first, the quality of the approximate process implemented remains comparable to other conditional operations commonly performed (for 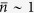)^11^,^13^,^14^,^34^,^35^. Remarkably, the fidelity for coherent states depends on the relative phase between the state and the operator. We have indeed verified that 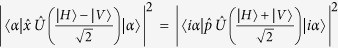 in all the reported range. Moreover, we observe that conditional process implemented on input states presenting a long tail in the photon number distribution, such as single-mode squeezed states, deviate more from the ideal targeted operation.

**Figure 3:**
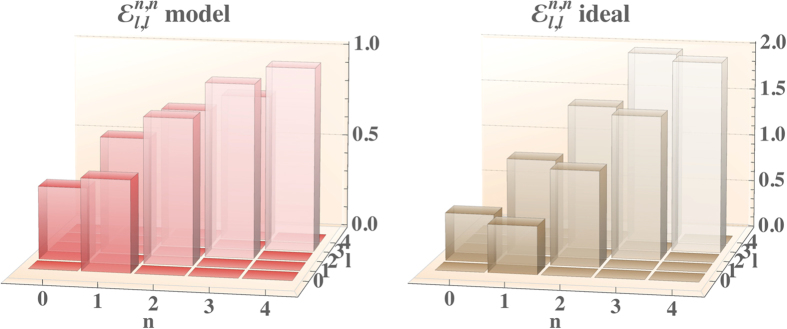
Diagonal part of the process tensor 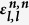 for both the modelled process Eq. (2) with *t* = 0.95 (left), and the ideal 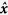 operation (right). Notice that, for ease of comparison, the modelled process tensor is rescaled by an overall factor 2/*r*^2^ due to the probabilistic nature of the process.

This occurrence can be understood by referring to the explicit form of the process tensor 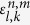, where each term is defined as the probability amplitude for 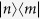 being transformed into 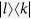
40,41. In Fig. 3, our results for the process tensor associated with 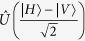 are compared with the ideal case 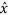. When observing the diagonal terms that govern the transfer of populations, 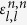, one observes that the main departure is the attenuation factor *C*^−(*n*+1)^*t*^*n*−1^, which is also responsible for the imbalance between the addition and subtraction terms.

### Robustness to experimental inefficiencies

Two main technical challenges must be met to achieve the proposed scheme. The first is associated with obtaining high-quality mode matching between signal and idler modes of the OPA^34^,^35^. This can be achieved by appropriately choosing the nonlinear interaction medium based upon its dispersion properties, and similarly well-chosen operating wavelength region to achieve degenerate phase matching associated with the photon addition process^42^,^43^,^44^,^45^. The second technical challenge to overcome is the limited detection efficiency of the heralding detectors. Numerical analysis of this effect has been examined for the process tensor 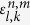, including the first-order corrections. However, we imagine to focus on operation in the high-efficiency regime, which has been achieved in recent demonstrations^46^,^47^,^48^.

**Figure 4:**
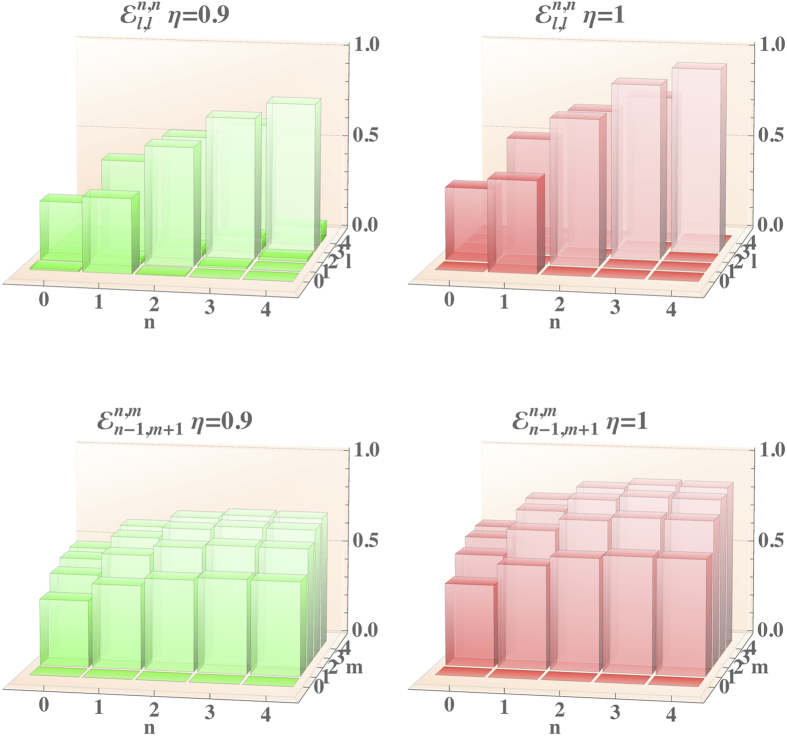
Comparison of the process tensor at different values of quantum efficiency of the detectors *η*, assumed equal for all involved. Top: diagonal part of the process tensor 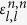. Bottom: section representative of the coherence transfer 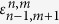. For ease of comparison, the modelled process tensor is rescaled by an overall factor 4/*r*^2^ due to the probabilistic nature of the process.

The results of this analysis are summarised in Fig. 4, where we show a comparison of the process tensors for two values of the detection efficiency of the photon-number resolving detectors, *η* = 0.9 and *η* = 1 for the programme 
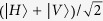
; full details on the form of the tensors are available in the Supplementary Information. For clarity of presentation, only a single heralding detection event associated with 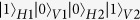 is considered, with the other events giving qualitatively similar results.

**Figure 5:**
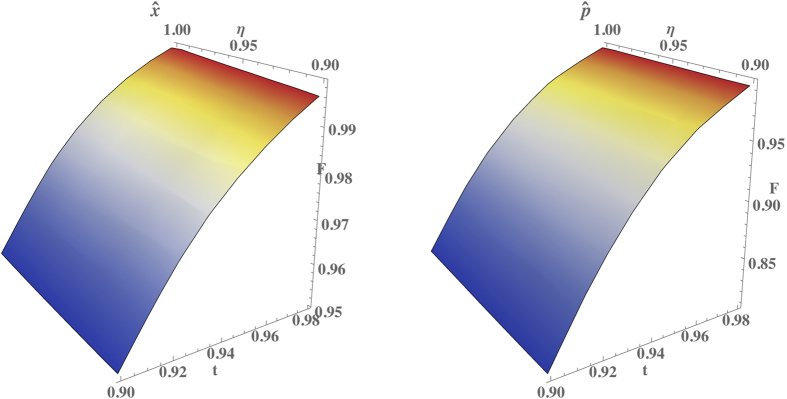
Comparison of fidelities between the ideal and modelled outputs for coherent states inputs 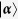 with *α* = 1 as a function of the transmission coefficient *t* and the detection efficiency *η* for 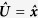 and 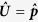.

The diagonal elements reveal that the attenuation at high photon number is more pronounced, since the inefficiency mainly results in the persistence of some population in original level 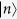. A minor effect consists in the transfer of population from 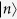 to 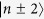, in analogy with the results in Ref. 49. Further insight is provided by observing the terms governing the coherence between photon-number components of the output state. The inefficiency of the detection process induces an overall attenuation of the off-diagonal coherence terms. In fact, the detection can not discriminate perfectly between different photon-number states, resulting in an incoherent mixture of distinct contributions. This is similar to the heralding effects on conditional operations found recently in Fock-state filtration^50^. The sheer effect is a reduction of the maximal photon number at which a satisfactory fidelity can be found with respect the results reported in Fig. 2. This effect is captured more quantitatively in Fig. 5, in which we show the fidelities between targeted and modelled outputs for coherent state inputs at moderate average photon number 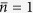. The protocol is resistant to small detection loss, *η* > 0.9 for 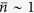, although it should be observed that these constraints will affect the overall success probability (see Supplementary Information).

## Discussion

We have presented a scheme for controlling the quantum state of a travelling light field conditioned on the logical state of a single optical qubit, which acts as a programme for a processing device. Our analysis highlights that a satisfactory fidelity, in excess of 95% can be achieved for a large class of states, in particular coherent states 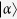 and cat states 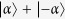. We also investigate the effect of imperfect detection on the process, which reveal technical challenges for realistic devices and the input photon number limit threshold for reasonable process fidelity with the target operation. Continued progress in detection technology^46^,^47^,^48^ and quantum light sources^45^,^51^ will allow implementation of this protocol in the near future.

The present protocol could likely have application in implementing an interface between qubits and mesoscopic systems, such as optomechanical platforms^52^ or atomic ensembles^53^. On the one hand, our scheme delivers a single-mode continuous-variable coding of the original qubit. In the presence of Gaussian light-matter entanglement, which can be produced in these mesoscopic systems, an unconditional teleportation protocol can realise an efficient mapping of the state^54^. On the other, the controlled operation can be used to performed controlled non-Gaussian transformation, when acting upon the entangled light, with the possibility of enhancing or restoring the level of entanglement^55^. A more ambitious programme might look into the direct coupling of the light field to the mesoscopic field, for instance by using a stimulated Raman field^56^ in a memory, or stimulated second-harmonic generation in toroids^57^, as a strategy for producing controllable nonGaussian entanglement. At present, all these operation still require the engineering of mesoscopic systems with an acceptance bandwidth large enough to allow pulsed operation, a task which is under intense development^58^,^59^,^60^. In general, this interface might allow for more flexible control of quantum light, towards the investigation and the exploitation of micro-macro quantum correlations^61^,^62^,^63^.

## Additional Information

**How to cite this article**: Barbieri, M. *et al.* Qubit-Programmable Operations on Quantum Light Fields. *Sci. Rep.* **5**, 15125; doi: 10.1038/srep15125 (2015).

## Supplementary Material

Supplementary Information
