# Supplementary material for: Qubit-Programmable Operations on Quantum Light Fields
Source: Sci Rep. 2015 Oct 15;5:15125. doi: 10.1038/srep15125 (PMC4606785; doi:10.1038/srep15125)
Supplement: Supplementary Information [file srep15125-s1.pdf]

Supplemental Material on "Qubit-Programmable Operations on Quantum Light Fields"

Marco Barbieri[1], Nicolò Spagnolo[2], Franck Ferreyrol[3], Rémi Blandino[4], Brian J. Smith[5], and Rosa Tualle-Brouri[6]

[1]*Dipartimento di Scienze, Università degli Studi Roma Tre, Rome, Italy*

[2]*Dipartimento di Fisica, Sapienza Università di Roma, Rome, Italy*

[3]*Laboratoire Photonique, Numérique et Nanostructures, Institut d'Optique, CNRS and Université de Bordeaux, Talence, France*

[4]*Centre for Quantum Computation and Communication Technology, School of Mathematics and Physics, University of Queensland, St Lucia, Queensland 4072, Australia*

[5]*Clarendon Laboratory, Department of Physics, University of Oxford, United Kingdom and*

[6]*Laboratoire Charles Fabry, Institut d'Optique, CNRS and Université Paris-Sud, Palaiseau, France and Institut Universitaire de France, Paris, France*

I. ANALYSIS OF THE IDEAL DEVICE

We start our analysis of the qubit-programmable operation with the amplification in the Optical Parametric Amplifier (OPA). Its action is described by the operator:

$$e^{g(\hat{a}_0^\dagger\hat{a}_1^\dagger+\hat{a}_0\hat{a}_1)} = e^{\Gamma\hat{a}_0^\dagger\hat{a}_1^\dagger}C^{-(\hat{n}_0+\hat{n}_1+1)}e^{-\Gamma\hat{a}_0\hat{a}_1}, \tag{1}$$

where $\Gamma = \tanh g$, and $C = \cosh g$. By acting with the operator (1) on the input $|n\rangle_0 |0\rangle_1$, we find:

$$e^{g(\hat{a}_0^\dagger\hat{a}_1^\dagger+\hat{a}_0\hat{a}_1)} |n\rangle_0 |0\rangle_2 = e^{\Gamma\hat{a}_0^\dagger\hat{a}_1^\dagger}C^{-(n+1)} |n\rangle_0 |0\rangle_1 . \tag{2}$$

We develop the exponential as a power series:

$$\frac{C^{-(n+1)}}{\sqrt{n!}}\sum_{j=0}^{\infty}\frac{\Gamma^j}{j!}\left(\hat{a}_0^\dagger\right)^{n+j}\left(\hat{a}_1^\dagger\right)^{j}|0\rangle_0|0\rangle_1 \tag{3}$$

which describes the output from the OPA. Subtraction is implemented by means of a beam splitter (BS) with transmissivity $t \simeq 1$, and reflectivity $r \leq 1$, which couples the input mode 0 with a vacuum mode 2:

$$\frac{C^{-(n+1)}}{\sqrt{n!}}\sum_{j=0}^{\infty}\frac{\Gamma^j}{j!}\sum_{k=0}^{n+j}t^{n+j-k}r^k\left(\hat{a}_0^\dagger\right)^{n+j-k}\left(\hat{a}_1^\dagger\right)^{j}\left(\hat{a}_2^\dagger\right)^{k}|0\rangle_0|0\rangle_1|0\rangle_2 \tag{4}$$

Mode 1 and mode 2 are superposed on a single spatial mode, by mapping mode 2 into its horizontal (H) polarisation, and mode 1 into the vertical (V) polarisation. These modes are denoted as $H1$ and $V1$

We now add the qubit $h|H\rangle + v|V\rangle$, which is written in the dual-rail representation as:

$$\left(h\hat{a}_{H2}^\dagger + v\hat{a}_{V2}^\dagger\right)|0\rangle_{H2}|0\rangle_{V2} \tag{5}$$

Spatial interference between mode 1 and the qubit mode is realised on a symmetric BS:

$$\begin{aligned}\hat{a}_{H1}^\dagger &= \frac{\hat{b}_{H1}^\dagger+\hat{b}_{H2}^\dagger}{\sqrt{2}}\\ \hat{a}_{H2}^\dagger &= \frac{\hat{b}_{H2}^\dagger-\hat{b}_{H1}^\dagger}{\sqrt{2}}\end{aligned} \tag{6}$$

and the transform for the V-polarisation are defined analogously.

These formulae allow us to study the interference between the output state (4) with the qbit (5); for the sake of simplicity, we isolate the term $\left(\hat{a}_{H1}^\dagger\right)^k\left(\hat{a}_{v1}^\dagger\right)^j\cdot\left(h\hat{a}_{H2}^\dagger+v\hat{a}_{V2}^\dagger\right)$, which is transformed by the action of the BS (6) as

$$\begin{aligned}\left(\frac{1}{\sqrt{2}}\right)^{j+k}\sum_{x=0}^{j}\sum_{y=0}^{k}h\left(\left(\hat{b}_{H1}^\dagger\right)^y\left(\hat{b}_{H2}^\dagger\right)^{k-y+1}\left(\hat{b}_{V1}^\dagger\right)^x\left(\hat{b}_{V2}^\dagger\right)^{j-x}-\left(\hat{b}_{H1}^\dagger\right)^{y+1}\left(\hat{b}_{H2}^\dagger\right)^{k-y}\left(\hat{b}_{V1}^\dagger\right)^x\left(\hat{b}_{V2}^\dagger\right)^{j-x}\right)+\\ +v\left(\left(\hat{b}_{H1}^\dagger\right)^y\left(\hat{b}_{H2}^\dagger\right)^{k-y}\left(\hat{b}_{V1}^\dagger\right)^x\left(\hat{b}_{V2}^\dagger\right)^{j-x+1}-\left(\hat{b}_{H1}^\dagger\right)^y\left(\hat{b}_{H2}^\dagger\right)^{k-y}\left(\hat{b}_{V1}^\dagger\right)^{x+1}\left(\hat{b}_{V2}^\dagger\right)^{j-x}\right)\end{aligned} \tag{7}$$

The indexes k, y, j, x must be fixed in such a way to give a non-zero product with $\langle 1|_{H1}\langle 0|_{H2}\langle 0|_{V1}\langle 1|_{V2}$. For this, it is required that either $k{=}y{=}0, j{=}1, x{=}0$ or $j{=}x{=}0, k{=}1, y{=}1$; the two index set correspond to the second term (in $-h$) and to the third term (in v), respectively. If we now substitute these indexes in Eq. (4), we obtain:

$$\begin{aligned}&\frac{C^{-(n+1)}t^{n-1}}{2\sqrt{n!}}\left(vrn\left(\hat{a}_0^\dagger\right)^{n-1}-h\Gamma t^2\left(\hat{a}_0^\dagger\right)^{n+1}\right)|0\rangle_0\\ &=\frac{C^{-(n+1)}t^{n-1}}{2}\left(vr\hat{a}_0-h\Gamma t^2\hat{a}_0^\dagger\right)|n\rangle_0\end{aligned} \tag{8}$$

The product with $\langle 0|_{H1}\langle 1|_{H2}\langle 1|_{V1}\langle 0|_{V2}$ delivers a similar expression.

Eq. (8) allows us to write explicitly the tensor process of the qubit-programmable operation. A generic quantum process from an input state ϱ to an output state ϱ' is described by a linear map $\varrho' = \mathcal{E}(\varrho)$. The action of the map $\mathcal{E}$ in the Fock basis can be represented as:

$$\varrho'_{l,k} = [\mathcal{E}(\varrho)]_{l,k} = \sum_{n,m} \mathcal{E}^{n,m}_{l,k}\, \varrho_{n,m}, \tag{9}$$

where $\mathcal{E}^{n,m}_{l,k}$ is the tensor process. The diagonal elements $\mathcal{E}^{n,n}_{l,l}$ of the tensor represent the transfer of population from the input to the output, while the remaining elements describe the evolution of coherence terms. The tensor $\mathcal{E}^{n,m}_{l,k}$ for the process described in Eq. (8) can be written as:

$$\begin{aligned}\mathcal{E}^{n,m}_{l,k} = C^{-m-n-2} t^{m+n-2} \Big(& v^2\sqrt{nm}\,\delta_{l,n-1}\delta_{k,m-1} - hv\sqrt{(n+1)m}\,\delta_{l,n+1}\delta_{k,m-1} + \\ & -vh\sqrt{n,m+1}\,\delta_{l,n-1}\delta_{k,m+1} + h^2\sqrt{(n+1)(m+1)}\,\delta_{l,n+1}\delta_{k,m+1}\Big)\end{aligned} \tag{10}$$

II. ANALYSIS WITH NON-IDEAL DETECTORS

We calculate the next-order correction when we introduce loss before the detectors. We assume that the efficiency η is the same for all the devices, and that these retain their number-resolving ability in full. We isolate a single term in Eq. (7), which we write in the general form

$$\left(\hat{b}^\dagger_{H1}\right)^\alpha \left(\hat{b}^\dagger_{H2}\right)^\beta \left(\hat{b}^\dagger_{V1}\right)^\gamma \left(\hat{b}^\dagger_{V2}\right)^\delta. \tag{11}$$

The effect of loss is modelled by a beam splitter of transmissivity $\sqrt{\eta}$, and we denote the loss modes as $\hat{l}^\dagger_{H1}$, and so on. The expression is modified as

$$\alpha\delta\eta(1-\eta)^{\frac{\alpha+\beta+\gamma+\delta-2}{2}}\, \hat{b}^\dagger_{H1}\left(\hat{l}^\dagger_{H1}\right)^{\alpha-1}\left(\hat{l}^\dagger_{H2}\right)^{\beta}\left(\hat{l}^\dagger_{V1}\right)^{\gamma}\hat{b}^\dagger_{V2}\left(\hat{l}^\dagger_{V2}\right)^{\delta-1} \tag{12}$$

where we selected the useful post-selection events with a single photon each on the $H1$ and $V2$ modes, and none on the $H2$ and the $V1$ modes. The partial trace on the loss modes lead to a pre-factor

$$\alpha\delta\eta(1-\eta)^{\frac{\alpha+\beta+\gamma+\delta-2}{2}}\sqrt{(\alpha-1)!}\sqrt{\beta!}\sqrt{\gamma!}\sqrt{(\delta-1)!} \tag{13}$$

which takes into account the probability amplitude associated to each event.

We consider the events with one photon in excess with respect the desired number: $|2\rangle_{H1}|0\rangle_{H2}|0\rangle_{V1}|1\rangle_{V2}$, $|1\rangle_{H1}|1\rangle_{H2}|0\rangle_{V1}|1\rangle_{V2}$, $|1\rangle_{H1}|0\rangle_{H2}|1\rangle_{V1}|1\rangle_{V2}$, and $|1\rangle_{H1}|0\rangle_{H2}|0\rangle_{V1}|2\rangle_{V2}$, *i.e.*, we insert in Eq.(12) and Eq.(13), α=2, β=0, γ=0, δ=1, and so on, excluding the cases with no physical meaning for the indexes k, y, j, x. The results are summarised in the Table, where the sets of unphysical indexes are written in red.

These indexes deliver the corrections to the leading term (8) (which now appears with an overall probability amplitude η):

$$\text{for 2,0,0,1,}\qquad \frac{\eta\sqrt{1-\eta}}{\sqrt{2}}C^{-(n+1)}t^{n-2}r\left(-ht^2\Gamma(\hat{n}_0+1)+\frac{vr}{2}\hat{a}_0^2\right)|n\rangle_0\,, \tag{14}$$

$$\text{for 1,1,0,1,}\qquad \frac{\eta\sqrt{1-\eta}}{4\sqrt{2}}C^{-(n+1)}t^{n-2}r^2v\,\hat{a}_0^2\,|n\rangle_0\,, \tag{15}$$

$$\text{for 1,0,1,1,}\qquad -\frac{\eta\sqrt{1-\eta}}{4\sqrt{2}}C^{-(n+1)}t^{n+2}\Gamma^2h\left(\hat{a}_0^\dagger\right)^2|n\rangle_0\,, \tag{16}$$

$$\text{for 1,0,0,2,}\qquad \frac{\eta\sqrt{1-\eta}}{\sqrt{2}}C^{-(n+1)}t^{n}\Gamma\left(vr(\hat{n}_0+1)-\frac{ht^2\Gamma}{2}\left(\hat{a}_0^\dagger\right)^2\right)|n\rangle_0\,. \tag{17}$$

$(\alpha,\beta,\gamma,\delta)$=	(2,0,0,1)	(1,1,0,1)	(1,0,0,1)	(1,0,0,2)
y=α<br>k-y+1=β<br>x=γ<br>j-x=δ	y=2<br>k=1<br>x=0<br>j=1	y=1<br>k=1<br>x=0<br>j=1	y=1<br>k=0<br>x=1<br>j=2	y=1<br>k=0<br>x=0<br>j=2
y+1=α<br>k-y=β<br>x=γ<br>j-x=δ	y=1<br>k=1<br>x=0<br>j=1	y=0<br>k=1<br>x=0<br>j=1	y=0<br>k=0<br>x=1<br>j=2	y=0<br>k=0<br>x=0<br>j=2
y=α<br>k-y=β<br>x+1=γ<br>j-x=δ	y=2<br>k=2<br>x=-1<br>j=0	y=1<br>k=2<br>x=-1<br>j=0	y=1<br>k=1<br>x=0<br>j=1	y=1<br>k=1<br>x=-1<br>j=1
y=α<br>k-y=β<br>x+1=γ<br>j-x=δ	y=2<br>k=2<br>x=0<br>j=0	y=1<br>k=2<br>x=0<br>j=0	y=1<br>k=1<br>x=1<br>j=1	y=1<br>k=1<br>x=0<br>j=1
